# Phylogenetic analysis of HIV-1 shows frequent cross-country transmission and local population expansions

**DOI:** 10.1093/ve/veab055

**Published:** 2021-06-09

**Authors:** Marc Bennedbæk, Anna Zhukova, Man-Hung Eric Tang, Jaclyn Bennet, Paula Munderi, Kiat Ruxrungtham, Magnus Gisslen, Michael Worobey, Jens D Lundgren, Rasmus L Marvig

**Affiliations:** Centre of Excellence for Health, Immunity and Infection (CHIP), Department of Infectious Diseases, Rigshospitalet, University of Copenhagen, Blegdamsvej 9, 2100 Copenhagen, Denmark; Unité Bioinformatique Evolutive, Hub Bioinformatique et Biostatistique, USR3756 (C3BI//DBC), Institut Pasteur and CNRS, 25-28 Rue du Dr Roux, 75015 Paris, France; Centre of Excellence for Health, Immunity and Infection (CHIP), Department of Infectious Diseases, Rigshospitalet, University of Copenhagen, Blegdamsvej 9, 2100 Copenhagen, Denmark; Clinical HIV Research Unit, Department of Internal Medicine, School of Clinical Medicine, Faculty of Health Sciences, University of Witwatersrand, 1 Jan Smuts Avenue, Braamfontein 2000, Johannesburg, South Africa; MRC Uganda Research Unit on AIDS, UVRI P.O.Box 49, Plot 51-59 Nakiwogo Road, Entebbe-Uganda; HIV-NAT, Thai Red Cross AIDS Research Center, and School of Global Health, Faculty Medicine, Chulalongkorn University, Chamchuri 5 Bld. 6th Fl., Phayathai Rd., Wangmai, Pathumwan Bangkok 10330, Thailand; Department of Infectious Diseases, Institute of Biomedicine, Sahlgrenska Academy, University of Gothenburg, Universitetsplatsen 1, 405 30 Gothenburg, Sweden; Department of Infectious Diseases, Region Västra Götaland, Sahlgrenska University Hospital, Universitetsplatsen 1, 405 30 Gothenburg, Sweden; Department of Ecology and Evolutionary Biology, University of Arizona, Biological Sciences West, Rm. 324 Tucson, AZ 85721, USA; Centre of Excellence for Health, Immunity and Infection (CHIP), Department of Infectious Diseases, Rigshospitalet, University of Copenhagen, Blegdamsvej 9, 2100 Copenhagen, Denmark; Department of Genomic Medicine, Rigshospitalet, Blegdamsvej 9, 2100 Copenhagen, Denmark

**Keywords:** HIV, Phylogenetics, Ancestral State Reconstruction, Transmission, Phylogeography

## Abstract

Understanding of pandemics depends on the characterization of pathogen collections from well-defined and demographically diverse cohorts. Since its emergence in Congo almost a century ago, Human Immunodeficiency Virus Type 1 (HIV-1) has geographically spread and genetically diversified into distinct viral subtypes. Phylogenetic analysis can be used to reconstruct the ancestry of the virus to better understand the origin and distribution of subtypes. We sequenced two 3.6-kb amplicons of HIV-1 genomes from 3,197 participants in a clinical trial with consistent and uniform sampling at sites across 35 countries and analyzed our data with another 2,632 genomes that comprehensively reflect the HIV-1 genetic diversity. We used maximum likelihood phylogenetic analysis coupled with geographical information to infer the state of ancestors. The majority of our sequenced genomes (*n* = 2,501) were either pure subtypes (A–D, F, and G) or CRF01_AE. The diversity and distribution of subtypes across geographical regions differed; USA showed the most homogenous subtype population, whereas African samples were most diverse. We delineated transmission of the four most prevalent subtypes in our dataset (A, B, C, and CRF01_AE), and our results suggest both continuous and frequent transmission of HIV-1 over country borders, as well as single transmission events being the seed of endemic population expansions. Overall, we show that coupling of genetic and geographical information of HIV-1 can be used to understand the origin and spread of pandemic pathogens.

## 1. Introduction

A high mutation rate, short generation time, lack of proofreading, and frequent recombination fuel HIV-1 molecular evolution with genetic variation (Castro-Nallar et al. 2012; Volz, Koelle, and Bedford 2013). This has resulted in the diversification of HIV-1 group M into nine distinct subtypes (A–D, F–H, J, and K). Recombination events between multiple subtypes have also resulted in new circulating recombinant forms (CRFs) of the virus. The prevalence of the different subtypes and CRFs differs markedly, with some more prevalent than others in specific geographic regions (Hemelaar et al. 2011, 2019). The divergence and expansion of the different subtypes of HIV-1 is elusive, as they are likely to have happened before the characterization of HIV-1 in 1983 (Gilbert et al. 2007).

Phylogenetics can be applied to infer the relatedness between HIV-1 sample genotypes, and geographical information contained in the genetic relationship can be used to infer the states of ancestral genotypes (Castro-Nallar et al. 2012). However, the retrospective nature of phylogenetics makes the approach dependent on the comprehensiveness of the sample collection, i.e. samples representing all genotypic states should ideally be included in the collection. In practice, it is not possible to sample all genotypic states, and especially it is difficult to include samples of ancestral genotypes only available from historical sample collections. Nonetheless, a few available historical collections of HIV-1 covering decades have been successfully sequenced and used to date origins of types of HIV-1 (Bletsa et al. 2019; Gryseels et al. 2020; Worobey et al. 2016). Beyond the geographical information contained in phylogenies, they can be analyzed with respect to other sample information, e.g. temporal, epidemiological, and demographic information, to further understand the observed genetic diversification and distribution of HIV-1 (Castro-Nallar et al. 2012).

Here, we sequenced HIV-1 genomes sampled from participants in the Strategic Timing of AntiRetroviral Treatment (START) trial (Group, Insight Start Study et al. 2015), which constitute a well-defined and demographically diverse cohort of ART-naïve HIV+ participants, and analyzed them together with a set of HIV-1 genomes defined by Los Alamos National Laboratory (LANL) to comprehensively reflect HIV-1 genetic diversity. With phylogenetic ancestry reconstruction, we used the sample collection to delineate and characterize the transmission of subtypes across countries.

## 2. Methods

### 2.1 Description of sample collection from START clinical trial

All participants are from the START trial, which is managed by the International Network for Strategic Initiatives in Global HIV Trials (INSIGHT). The study design and data collection plan for START have previously been reported (Group, Insight Start Study et al. 2015). A total of 4,685 antiretroviral therapy (ART)-naive participants from 35 countries (6 geographical regions) were included in the START study. The participants were enrolled between April 2009 and December 2013. Here, 3,785 participants with a viral load ≥1,000 cp/ml and with at least 2 baseline plasma samples available in the START biobank were included for HIV-1 genome sequencing.

### 2.2 Ethics

Samples included in this study were derived from participants who consented to the clinical trial, START (NCT00867048) (Group, Insight Start Study et al. 2015), run by INSIGHT. The study was approved by the institutional review board or ethics committee at each contributing center, and written informed consent was obtained from all participants. All informed consents were reviewed and approved by participant site ethics review committees.

### 2.3 Viral concentration and RNA extraction

Plasma samples were thawed at room temperature. Following this, 500 µl plasma was transferred to new RNAse-free tubes and centrifuged at 2,000×*g* for 15 minutes. The supernatant was then extracted and centrifuged at 21.000×*g* for 75 minutes, and 360 µl of the top supernatant was discarded. Viral RNA was extracted using QIAamp viral RNA extraction kit (Qiagen) on a QIAcube robot according to the manufacturer’s guidelines.

### 2.4 Reverse-transcription and amplification of viral RNA

Reverse-transcription polymerase chain reaction (RT-PCR) was used to amplify two amplicons of HIV-1 separately, covering positions 1485–5058 and 5967–9517 (later referred to as amplicons A and B) in the HXB2 genome sequence (GenBank accession number K03455), as described previously (primer sequences are listed in Supplementary Table S1) (Gall et al. 2012). The reverse-transcription and amplification were performed using SuperScript III One-Step RT-PCR System with Platinum Taq High Fidelity (Thermo Fisher Scientific) according to the manufacturer’s instructions with 10 µl viral RNA for each amplicon (Thermocycler settings are listed in Supplementary Table S7). The PCR products were purified using Ampure XP (Beckman Coulter) PCR purification according to the manufacturer’s instructions. The two amplicons were pooled for each sample prior to DNA library preparation.

### 2.5 Amplicon DNA library preparation and sequencing

Libraries of DNA from pooled amplicons were prepared using a Nextera XT (Illumina, San Diego, CA, USA) sample preparation kit to target an insert size of 300 nucleotides (nts). A modified protocol was used, in which input DNA and reagent use was halved, except for normalization of libraries, where 1.5× magnetic normalization beads were used. DNA libraries were sequenced on an Illumina MiSeq machine using a MiSeq 150-cycle V3 reagent kit (Illumina), producing 75-nt paired-end reads.

### 2.6 Removal of sequence readouts from human genome

Sequencing reads from each sample were aligned against the human reference genome sequence Genome Reference Consortium Human Build 37 patch release 13 (GRCh37.p13) using Bowtie2 version 2.2.8 (Langmead and Salzberg 2012) (option ‘-X 1000’) and only read pairs in which neither of the reads aligned to GRCh37.p13 were retained.

### 2.7 Alignment of reads against HXB2 reference to create consensus sequence

Cleaned reads from the previous step were aligned against the genome sequence of HIV-1 HXB2 (GenBank accession number K03455.1) with SSAHA2 version 2.5.3 (Ning, Cox, and Mullikin 2001) (option ‘-solexa’ and ‘-pair 11000’), and resulting sequence alignment map (SAM) files were converted to sorted binary alignment map (BAM) files using SAMtools 1.1 (Li et al. 2009). Insert size of mapped reads was on average 173 nts (interquartile range 123–216 nts) as determined with command ‘samtools stats [sorted BAM file]’. Consensus sequences of reads aligned within regions targeted by amplicons A and B were generated from sorted BAM files with the command ‘samtools mpileup -d 1000000 -uf [HXB2 reference sequence] [sorted BAM file] | bcftools call -c | vcfutils.pl vcf2fq’. Sample consensus sequences were trimmed to only contain the genomic position of the two amplicons, i.e. HXB2 reference genome positions 1485–5058 (amplicon length 3,574 nts) and 5967–9517 (amplicon length 3,551 nts). All samples (*n* = 3,197) for which a consensus sequence was determined for either 90 per cent of one of the amplicon regions or 50 per cent of both amplicon regions were included for further subtype and phylogenetic analysis.

### 2.8 HIV-1 subtyping

The consensus sequence of each sample was analyzed with REGA HIV-1 Subtyping Tool version 3 (Pineda-Pena et al. 2013). The output was manually inspected to check for the presence of subtype-specific sequences within the given consensus sequence. Shannon–Weaver and 1—Simpson diversity indexes were calculated using the vegan package in R (version 3.2.5). The diversity indexes were calculated based on the distribution of subtypes A, AB, B, BC, BF, C, and CRF01_AE across geographical regions.

### 2.9 Genome sequences from Los Alamos HIV sequence database

We downloaded publicly available HIV-1 genome sequences from Los Alamos HIV Sequence Database (LANL). We downloaded all genome sequences in LANL Filtered Web Alignment (https://www.hiv.lanl.gov/content/sequence/NEWALIGN/align.html) of subtypes A (A1–A6), B, C, D, F (F1 and F2), G, and CRF01_AE. The sequences were downloaded on 23 November 2020. The sequences were trimmed to match the START amplicons using MAFFT version 7.453 with the following command:

mafft --thread 26 --memsave --retree 1 --maxiterate 0 --add <LANL_filtered_web_aligment_fasta-file-with-sequences> --keeplength <K03455_subset_to_ amplicon_A_and_B> <LANL_filtered_web_alignment_aligned_and_subset_to_amplicon_A_and_B>. The aligned sequences were combined with the STARTsequences.

### 2.10 Maximum likelihood phylogenetic reconstruction

**Figure 1.**
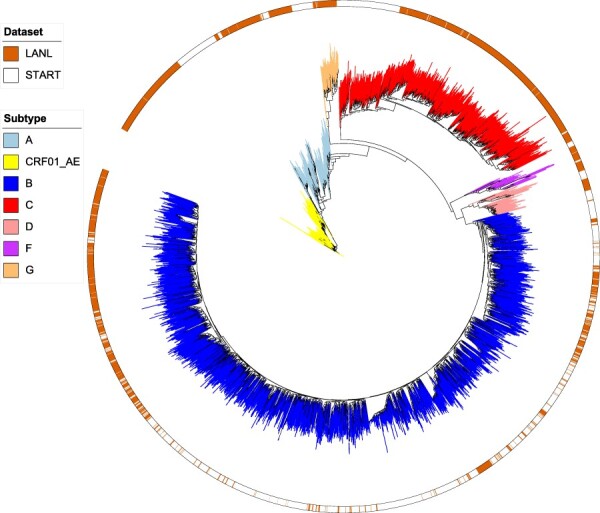
Maximum likelihood phylogenetic tree of all 5,133 samples in the combined START and LANL datasets that were defined as pure subtypes (A–D, F, and G) or subtype CRF01_AE. The branches are colored according to subtype. The outer color strip indicates if the samples are from either START or LANL Filtered Web alignment.

A maximum likelihood tree was constructed using ExaML (Exascale Maximum Likelihood) version 3.0.16 that uses the RAxML search algorithm (Kozlov, Aberer, and Stamatakis 2015). The sequences of pure subtypes A, B, C, D, F, and G, as well as subtype CRF01_AE were used for tree construction. Recombination hampers phylogenetic analysis, so we focused our analysis of ancestry on subtypes A, B, C, and CRF01_AE, respectively (Felsenstein 2004). We used a generalized time-reversible model with a gamma distribution, and the analysis was parallelized using openmpi. The following three commands were used to produce the trees: (1) ‘raxmlHPC-AVX -y -m GTRGAMMA -p 12345 [sample consensus sequences in phylip format] -n StartingTree’, (2) ‘examl-OMP -s maximum_likelihood_tree.unpartitioned.binary -t RAxML_parsimonyTree.StartingTree -m GAMMA -n Tree1’, and (3) ‘examl-AVX -s maximum_likelihood_tree.unpartitioned.binary -t TreeSet -f E -m GAMMA -n T3uE’. The maximum likelihood trees were visualized using the Interactive Tree of Life online tool (Fig. 1) (Letunic and Bork 2019). Least-Squares Dating (LSD) was used to extract monophyletic clades containing subtypes A, B, C, and CRF01_AE, respectively, and produce separate subtype-specific rooted trees (To et al. 2016).

### 2.11 Reconstruction of ancestral states

Ancestral states were reconstructed using PastML 1.9.7 (Ishikawa et al. 2019). We used the rooted subtype-specific trees produced by LSD as input for PastML. The tips in the subtype-specific trees were annotated to country. Ancestral state reconstruction and visualization in PastML were run with the following command: ‘pastml --tree (International HIV Controllers Study 2010) --data [metadata] --columns [metadata column] --name_column [metadata column] --tip_size_threshold 15 --html_compressed [compressed_tree] --html [uncompressed_tree] --prediction_method MPPA --model F81’. We used the PastML utility script ‘calculate_changes.py’ (https://github.com/evolbioinfo/pastml/blob/master/pastml/utilities/calculate_changes.py) to count the inferred events of transmission between countries with the following command: calculate_changes.py—tree (International HIV Controllers Study 2010)—acr [file with combined ancestral states output from pastml]—columns Country—out_log [log file].

Ancestral states were reconstructed with PastML using an F81-like model, which generalizes states to the 4-state F81 model for nucleotide substitution (Felsenstein 1981). Under F81-like model, migration rate from a state i (e.g. location) to a different state j (i != j) is proportional to the equilibrium frequency of j, πj. In addition to the state equilibrium frequencies, PastML optimizes the rescaling factor (analogous to mutation rate under strict molecular clock), which is applied to all the tree branches and represents the average number of character changes per branch unit (e.g. year for dated trees).

We assessed the robustness of the ancestral state reconstruction by shuffling tip state annotations prior to PastML analysis. The random shuffling analysis was repeated three times for each subtype.

## 3. Results

### 3.1 Genotyping of HIV-1 from the START clinical trial

We obtained HIV+ plasma samples from 3,785 START trial participants with a viral load ≥1,000 copies/ml. For each sample, we sequenced two 3.6-kb amplicons of the HIV-1 genome that cover partial *gag* (54 per cent) and *pol* (99 per cent), and full the *env* gene, respectively. While we achieved an average sequencing coverage of the two HIV-1 genomic regions of 7,188-fold (median 5,334-fold) and 1,061-fold (median 303-fold), respectively, sequencing success varied across both samples and genomic regions. For further analysis, we only included those 3,197 samples for which sequence information was available for at least 90 per cent of either of the genomic regions or at least 50 per cent of both genomic regions.

We analyzed each of the 3,197 genomes with REGA HIV-1 Subtyping Tool (Pineda-Pena et al.) to identify subtype-specific sequences, and we assigned the samples to be either a pure subtype (*n* = 2,354 samples distributed on subtypes A–D, F, and G), or a recombinant subtype, in cases where the genome showed the presence of sequences specific to >1 pure subtypes (*n* = 843 samples). We did not detail the recombinants down to specific CRF references, except for CRF01_AE (*n* = 147). For example, if a genome showed to contain sequence identities specific to both subtype C and D, respectively, it was denoted as subtype ‘CD’ without further detailing of genomic mosaic structure defined by recombinant breakpoints.

### 3.2 Combined phylogenetic analysis of data from START and LANL

We combined our START sequences of pure subtypes (A, B, C, D, F, and G) and CRF01_AE (*n* = 2,501 sequences) with all sequences of the same subtypes from the LANL Filtered Web Alignment (*n* = 2,632) that consist of sequences that represent the fullest spectrum (diversity) of sequences in LANL (Table 1).

**Table 1.** Number of samples for subtypes A, B, C, D, F, G, and CRF01_AE in START and LANL datasets.

Subtype	START	LANL
A	106	231
B	1,959	1,196
C	229	720
D	35	71
F	20	46
G	5	76
CRF01_AE	147	292
Total	2,501	2,632

The LANL Filtered Web Alignment sequences spanned seventy-two countries (eight samples have no country information) of which twenty-four countries overlapped with the thirty-five countries in START (Supplementary Tables S2–S3). Sequences from START were sampled between April 2009 and December 2013 (Supplementary Fig. S1). The majority (94 per cent) of sequences in LANL Filtered Web Alignment were from samples after 1995, albeit the earliest samples dated back to 1979 (Supplementary Fig. S1).

We constructed a maximum likelihood phylogeny of all samples in the combined dataset consisting of subtypes A, B, C, D, F, G, and CRF01_AE (*n* = 5,133; Fig. 1). The phylogeny confirmed that all samples of each subtype clustered as monophyletic groups, i.e. there was concordance between the assigned subtypes and the evolutionary relationship of samples. Subtypes A, B, C, and CRF01_AE were the most frequent subtypes in both the START and the LANL datasets, and our further analysis was focused on extracted monophyletic clades for each of these four subtypes (i.e. a rooted phylogenetic tree for each subtype; Supplementary Figs S2–S5).

### 3.3 Origin and cross-country transmission of subtype B

Subtype B samples from LANL Filtered Web Alignment (*n* = 1,196) encompassed 43 countries of which 15 countries overlapped with the 32 countries with subtype B samples from START (*n* = 1,959; Supplementary Tables S2–S3).

**Figure 2.**
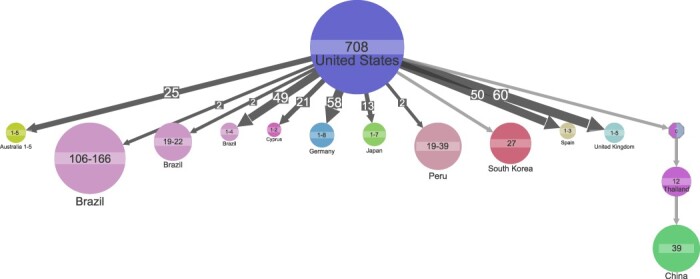
Ancestral state reconstruction of 3,155 subtype B samples. Ancestral state reconstruction of samples is shown for country origin. Circles denote genetic clusters of samples with the same state. The state and sample size of clusters are indicated for each circle. An arrow between two circles denotes events of transmission from the top cluster to the bottom cluster. The size and the number on top of the arrows indicate that the arrows represent multiple transmission events leading to clusters of similar sizes. Clusters with a ‘0’ and multiple colors indicate that several corresponding states have similar marginal probabilities. The lowest marginal probability for resolved clusters shown is 74 per cent. Clusters of size less than 19 are hidden to improve readability.

We annotated the tips (samples) of the subtype B phylogenetic tree according to country of origin to infer the ancestral states (Fig. 2). We found that our collection of subtype B samples emerged in the USA (marginal probability 89 per cent). To assess if our ancestral state estimates were a result of genuine structure in the data or due to collection skewness (subtype B samples were in general most often observed in the USA (25 per cent)).

We repeated the ancestral reconstruction with geographical annotation randomly shuffled between samples (Firth et al. 2010). Random shuffling of country annotation led to the estimate of ancestral state being unknown (Supplementary Fig. S6). As such, the random shuffling of annotation supported the estimate that the ancestral state was in the USA and was a result of genuine structure in the data.

We used ancestral state reconstruction to infer events of transmission between countries to explain the observed country of origin of our samples (Fig. 2). We inferred 530 events of transmission of subtype B from the USA to Europe or Australia, and all transmission events led to clusters of one to eight samples in the recipient country. We also inferred sixty-one transmissions of subtype B back again to the USA, Europe, or Australia.

While we also inferred 179 transmission events from the USA to Latin America leading to small clusters represented by one to four samples, six transmission events from the USA to Latin America distinguished themselves as they led to larger clusters of 19, 19, 22, 39, 106, and 166 samples, respectively (Fig. 2). Five of these clusters included samples from both START and LANL datasets.

We found that transmission from the USA led to a large cluster represented by twenty-seven samples from South Korea (Fig. 2). Also, we found a cluster of twelve samples in Thailand to originate from the USA or Malaysia, and that the Thailand cluster seeded a large cluster in China represented by thirty-nine samples (Fig. 2).

While subtype B originally emerged in Central Africa (Gilbert et al. 2007), ancestral reconstruction estimates that all African subtype B samples in our collection are the results of introduction of subtype B from other geographical regions back to Africa.

### 3.4 Origin and cross-country transmission of subtype C

**Figure 3.**
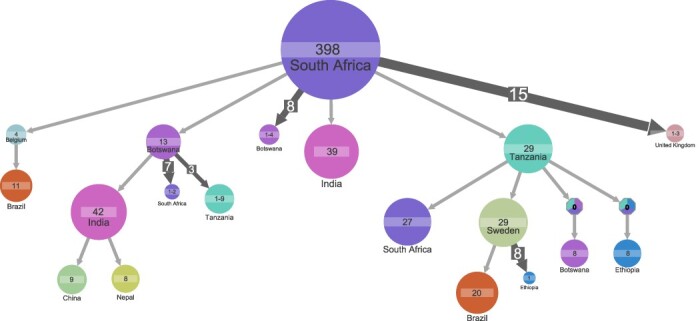
Ancestral state reconstruction of 949 subtype C samples. Ancestral state reconstruction of samples is shown for country origin. Circles denote genetic clusters of samples from the sample country. The country and sample size of clusters are indicated for each circle. An arrow between two circles denotes events of transmission from the top cluster to the bottom cluster. Clusters with a ‘0’ and multiple colors indicate that several corresponding states have similar marginal probabilities. The lowest marginal probability for resolved clusters shown is 82 per cent. The size and the number on top of the arrows indicate that the arrow represents multiple transmission events leading to clusters of similar sizes. Clusters of size less than 8 are hidden to improve readability.

The second most prevalent subtype within our combined dataset was subtype C (*n* = 949). Subtype C samples from LANL Filtered Web Alignment (*n* = 720) encompassed 30 countries of which 10 countries overlapped with the 21 countries with subtype C samples from START (*n* = 229) (Supplementary Tables S2–S3). Ancestral state reconstruction estimated the ancestor of subtype C samples to be in South Africa (marginal probability 99 per cent), and root cluster encompassed 398 samples across both datasets (Fig. 3). The country of the ancestor became unknown when the country annotation was randomly shuffled (Supplementary Fig. S7). Accordingly, the annotation shuffling supported the estimation of the subtype C ancestral state to be in South Africa and was the outcome of genuine structure in the data.

From the ancestral cluster in South Africa, we inferred four events of transmission that led to clusters represented by >10 samples: (1) transmission to a thirty-nine-sample cluster in India; (2) transmission to a twenty-nine-sample cluster in Tanzania with transmission onward via Sweden (cluster with twenty-nine samples) to Brazil (cluster with twenty samples) and from Tanzania back to South African cluster represented by twenty-seven samples; (3) transmission to a four-sample cluster in Belgium with transmission onward to Brazil (cluster with eleven samples); and (4) transmission to a thirteen-sample cluster in Botswana with transmission onward to an Indian cluster (forty-two samples) (Fig. 3). The largest Brazilian cluster included samples from both START and LANL datasets.

### 3.5 Origin and cross-country transmission of subtype A

**Figure 4.**
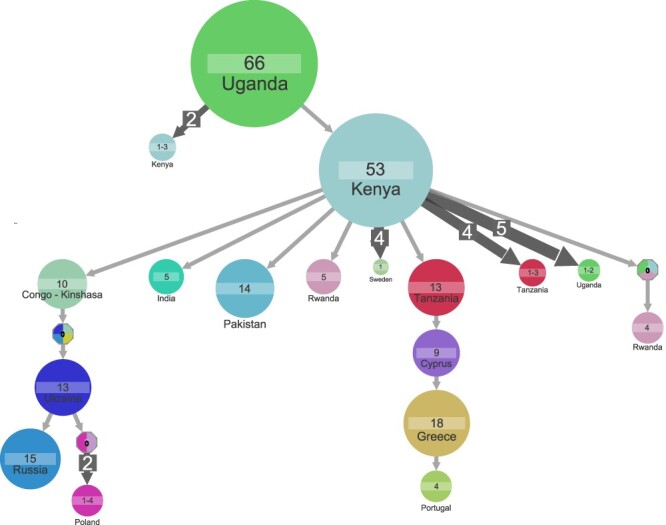
Ancestral state reconstruction of 337 subtype A samples. Ancestral state reconstruction of samples is shown for country origin. Circles denote genetic clusters of samples from the sample country. The country and sample size of clusters are indicated for each circle. An arrow between two circles denotes events of transmission from the top cluster to the bottom cluster. Clusters with a ‘0’ and multiple colors indicate that several corresponding states have similar marginal probabilities. The lowest marginal probability for resolved clusters shown is 75 per cent. The size and the number on top of the arrows indicate that the arrow represents multiple transmission events leading to clusters of similar sizes. Clusters of size less than 4 are hidden to improve readability.

START subtype A samples (*n* = 106) encompassed 12 countries, whereas LANL subtype A samples (*n* = 231) encompassed 22 countries of which 4 countries overlapped with START (Supplementary Tables S2–S3). The ancestor of subtype A samples was estimated to be in Uganda (marginal probability 99.8 per cent), and the root cluster of sixty-six samples encompassed samples from both datasets (Fig. 4). Estimate of the ancestral state became unknown when the country annotation was randomly shuffled (Supplementary Fig. S8); thus, the shuffling of country annotation supports that the estimation of subtype A ancestral state to be in Uganda was the outcome of genuine structure in the data. From the ancestral cluster in Uganda, we inferred up to 11 transmissions leading to clusters of sizes 1–3 and a transmission leading to a large cluster in Kenya (53 samples) with onward transmission to clusters including a total of 184 samples (Fig. 4).

The large Kenyan cluster was the source of two introductions to Europe that led to clusters represented by a minimum of four samples: that is, we delineated clusters of transmission from the Kenyan cluster to (1) Tanzania (twelve samples) to Cyprus (nine samples) to Greece (eighteen samples) to Portugal (four samples) and (2) Congo (ten samples) with onward transmission to Ukraine (thirteen samples) that split into a Russian (fifteen samples) and a Polish cluster (four samples), respectively (Fig. 4). While both START and LANL datasets included Ugandan samples, the other before-mentioned countries were either unique to LANL (Congo, Ukraine, Russia, Poland, Tanzania, Cyprus) or START (Greece, Portugal) dataset, respectively.

### 3.6 Origin and cross-country transmission of subtype CRF01_AE

For subtype CRF01_AE, the LANL dataset encompassed samples (*n* = 292) from 14 countries of which three countries overlapped with the samples (*n* = 147) from 12 countries encompassed by the START dataset (Supplementary Tables S2–S3).

**Figure 5.**
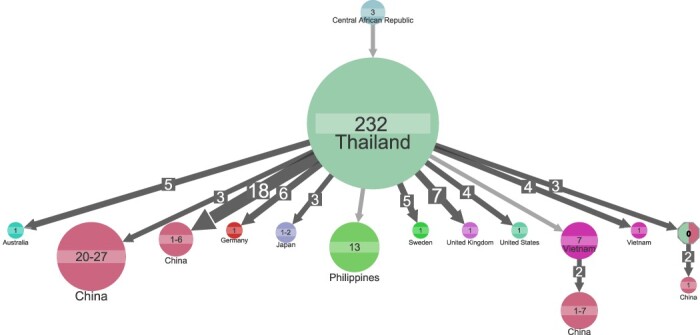
Ancestral state reconstruction of 439 subtype CRF01_AE samples. Ancestral state reconstruction of samples is shown for country origin. Circles denote genetic clusters of samples from the sample country. The country and sample size of clusters are indicated for each circle. An arrow between two circles denotes events of transmission from the top cluster to the bottom cluster. The lowest marginal probability for resolved clusters shown is 64 per cent. The size and the number on top of the arrows indicate that the arrow represents multiple transmission events leading to clusters of similar sizes. Clusters of size less than 4 are hidden to improve readability.

Most (234 of 439 samples) of subtype CRF01_AE samples were from Thailand, but while the majority (81 per cent) of subtype CRF01_AE samples in START dataset were from Thailand, subtype CRF01_AE samples in LANL dataset were more distributed in both China (42 per cent) and Thailand (39 per cent). The LANL subtype CRF01_AE dataset also included three samples taken in 1990 in the Central African Republic, and a cluster with these three samples placed the origin of CRF01_AE to be Central African Republic (marginal probability 64 per cent; Fig. 5). Estimate of the ancestral state became unknown when the country annotation was randomly shuffled (Supplementary Fig. S9); thus, the shuffling of country annotation supports that the estimation of subtype CRF01_AE ancestral state to be in Central African Republic was the outcome of genuine structure in the data.

A transmission cluster in Thailand (*n* = 232 samples, including samples from both LANL and START datasets) was the largest cluster identified for CRF01_AE. From Thailand, we inferred forty-two transmissions that led to small clusters outside Asia, whereas we found transmission from Thailand to other Asian countries that led to six large clusters of seven to twenty-seven samples (Fig. 5).

### 3.7 Subtype distribution across regions for START clinical trial samples

Finally, we investigated the distribution of subtypes across geographical regions for START samples. Seven subtypes (A, AB, B, BC, BF, C, and CRF01_AE) were represented by at least 100 samples in the START collection (Table 2). The seven subtypes showed differences in distribution across geographical regions (Pearson’s Chi-squared test with simulated *P*-value based on 2,000 replicates; *P*-value = 0.0005). Subtype B was the most dominant in Australia, Europe and Israel, Latin America, and the USA; subtype C was most dominant in Africa; and subtype CRF01_AE was most dominant in Asia (Table 2). The population of HIV-1 in the USA was the most homogenous (Shannon–Weaver index 0.11), whereas the African population was the most diverse (Shannon–Weaver index 1.52; Table 2).

**Table 2.** Distribution of HIV-1 subtypes across geographical regions for START dataset. All subtypes found in less than 100 samples are grouped as ‘Other’. Sequencing was not successful for 588 samples and these are shown as ‘ND’ (not determined). The bottom two rows show the Shannon–Weaver and 1—Simpson indexes of subtype diversity per region. Diversity indexes were calculated based on the distribution of subtypes A, AB, B, BC, BF, C, and CRF01_AE across geographical regions.

Subtype	Africa	Asia	Australia	Europe and Israel	USA	Latin America	Total
A	63 (9%)	0	0	43 (3%)	0	0	106 (3%)
AB	43 (6%)	19 (6%)	0	44 (3%)	2 (1%)	2 (0%)	110 (3%)
CRF01_AE	0	121 (41%)	5 (5%)	21 (2%)	0	0	147 (4%)
B	42 (6%)	23 (8%)	74 (78%)	888 (66%)	338 (85%)	594 (65%)	1,959 (52%)
BC	85 (12%)	8 (3%)	1 (1%)	22 (2%)	1 (0%)	23 (3%)	140 (4%)
BF	0	0	0	26 (2%)	1 (0%)	132 (14%)	159 (4%)
C	129 (18%)	40 (13%)	3 (3%)	40 (3%)	2 (1%)	15 (2%)	229 (6%)
Other	189 (26%)	19 (6%)	0	109 (8%)	5 (1%)	25 (3%)	347 (9%)
ND	180 (25%)	68 (23%)	12 (13%)	160 (12%)	48 (12%)	120 (13%)	588 (16%)
Total	731	298	95	1,353	397	911	3,785
Shannon–Weaver Index	1.52	1.22	0.44	0.79	0.11	0.70	−
1—Simpson index	0.76	0.61	0.20	0.32	0.03	0.37	−

## 4. Discussion

In this study, we sequenced HIV-1 genomes from 3,197 participants in a clinical trial sampling across 35 countries and analyzed our data with another 2,632 genomes that comprehensively reflect the HIV-1 genetic diversity.

We used the genetic information to delineate and characterize cross-country transmission of subtypes. Our transmission analysis of subtype B showed many parallel transmissions out from the USA that led to small transmission clusters in other regions (fifty-one countries). We also found evidence for transmission back again to the USA from twenty-nine different countries. We find that this supports a model of continuous and frequent transmission of subtype B over country borders.

While we in general found transmission to be identified by clusters represented by a few samples, transmission of subtype B from the USA to Latin America showed examples of single transmissions that led to large clusters. We suggest that this is indicative that the HIV-1 population expansion within Latin America was local rather than being fueled by new transmissions from other regions. A similar indication of local clonal expansion was observed for subtype C for which an ancestral population in South Africa led to large clusters in India. This is in agreement with previous reports that the introduction of subtype C to India occurred a limited number of times (Neogi et al. 2012). We found the root of subtype CRF01_AE to be in central Africa, which is also the known origin for this subtype (Gao et al. 1996; Murphy et al. 1993). Also, the distribution of subtype CRF01_AE in the START clinical trial confirms that subtype CRF01_AE is endemic in Asia (Angelis et al. 2015), and while we found frequent transmissions to Europe and Australia, our phylogenetic analysis shows that there is no expansion of subtype CRF01_AE outside Asia.

A limitation of our analysis is that even though our sample collection includes 84 countries, many geographical regions are not well represented, and results from one country may not represent nearby countries, as there may exist large differences in subtype distribution between countries in the same region. This is illustrated by Greece that, unlike other European countries, had a high prevalence of subtype A in the START clinical trial. This agrees with a report on people living with HIV-1 in southwestern Greece that concludes subtype A has surpassed subtype B in new infections (Davanos et al. 2015), and we furthermore estimated that the Greek cluster of subtype A was transmitted from Uganda.

Also, we found that subtype B ancestral state was estimated to be in the USA; nonetheless, we note that, as we have no Caribbean samples in our collection, our analysis is not able to detect the suggested cryptic subtype B circulation in the Caribbean (Worobey et al. 2016).

We found it confirmatory that transmission clusters often included both START and LANL samples and as such both datasets both supported the existence of these clusters. While some coun tries were represented in both datasets, most countries were unique to each of the two datasets. In this regard, we found it confirmatory that we were able to delineate transmission over countries that were one after the other unique to one of the datasets. Finally, we also found it confirmatory that inferred cross-country transmission often followed the geographical position of countries, e.g. we observed a north-eastwards transmission of subtype C: South Africa to Botswana to India to China or Nepal. Nonetheless, while we found that the combined dataset showed to mutually support our findings, we note that analysis of combined datasets should be interpreted with caution as results may be confounded by general differences between the datasets, e.g. differences in sequence quality.

We took advantage of the START sampling scheme to investigate the distribution of subtypes across geographical regions during 2009–13. While we identified the majority of START samples be pure subtypes (A–D, F, and G), we found 26 per cent recombinant subtypes including more than >100 samples of recombinants AB, BC, BF, and CRF01_AE, respectively. This is in line with that recombinant forms of HIV-1 have been estimated to account for 18–20 per cent of infections worldwide (Buonaguro, Tornesello, and Buonaguro 2007; Hemelaar et al. 2011, 2019). It was beyond the scope of this study to perform a detailed characterization of the individual mosaics and their ancestry (except for CRF01_AE); nonetheless, we note that our observed subtype distributions confirm a high prevalence of subtypes BC and BF in Africa and Latin America, respectively (Carr et al. 2001; Melo, Jamal, and Zanotto 2012). Also, we found subtype AB in Uganda, Thailand, and Greece, but our sample collection does not cover Eastern Europe where an AB recombinant was first described in 1998 to circulate among injecting drug users in Eastern Europe (Liitsola et al. 1998), and further reports on distribution and transmission of AB recombinants remain scarce (Neogi et al. 2017).

Like other studies using the same amplicon-based sequencing approach (Cornelissen et al. 2017; Ratmann et al. 2017), we were limited by our ability to produce an evaluable result for all available START samples, and likewise, we observe that failure increased for the 3ʹ end of the genome and samples with low viral load. Also, our use of machine extraction as opposed to manual RNA extraction may affect our success rate (Cornelissen et al. 2017).

Finally, we note that our two datasets are fundamentally different in the sense that the START samples represent HIV-1 from ART-naïve clinical trial participants with CD4+ cell counts greater than 500 cells/mm^3^ (Group, Insight Start Study et al. 2015), whereas LANL samples were selected to represent the fullest spectrum of HIV-1 genetic diversity.

Overall, our data both extend current knowledge and exemplify the limitations in phylogeographic analysis, where inference of transmission is dependent on both representative geographical and genotypic sampling, and as such the estimated countries of ancestors should only be regarded as the best proxies, given not all countries are represented. Also, we note that our combined analysis encompasses eighty-four countries, but forty-four of these are represented by less than ten sequences.

In conclusion, we have used a combined dataset of HIV-1 genomes to present an analysis on the ancestry and transmission of HIV-1 across multiple viral subtypes and geographical regions. We envision that the presented data on HIV-1 genotypes can facilitate comparative genetic studies and be analyzed in concert with other information, e.g. markers of HIV pathogenesis, to further understand HIV-1.

## Supplementary Material

veab055_Supp

## Data Availability

All non-personally identifiable data will be made available to the readers of *Virus Evolution* Genome sequences and associated metadata have been deposited at Dryad repository: https://doi.org/10.5061/dryad.pnvx0k6k0.

## References

[R1] Angelis K. et al. (2015) ‘Global Dispersal Pattern of HIV Type 1 Subtype CRF01_AE: A Genetic Trace of Human Mobility Related to Heterosexual Sexual Activities Centralized in Southeast Asia’, *Journal of Infectious Diseases*, 211: 1735–44. 25512631 10.1093/infdis/jiu666

[R2] Bletsa M. et al. (2019) ‘Divergence Dating Using Mixed Effects Clock Modelling: An Application to HIV-1’, *Virus**Evolution*, 5: vez036. 10.1093/ve/vez036 PMC6830409 31720009

[R3] Buonaguro L., Tornesello M. L., and Buonaguro F. M. (2007) ‘Human Immunodeficiency Virus Type 1 Subtype Distribution in the Worldwide Epidemic: Pathogenetic and Therapeutic Implications’, *Journal of Virology*, 81: 10209–19. 17634242 10.1128/JVI.00872-07 PMC2045484

[R4] Carr J. K. et al. (2001) ‘Diverse BF Recombinants Have Spread Widely Since the Introduction of HIV-1 into South America’, *AIDS*, 15: F41–7. 11600844 10.1097/00002030-200110190-00002

[R5] Castro-Nallar E. et al. (2012) ‘The Evolution of HIV: Inferences Using Phylogenetics’, *Molecular Phylogenetics and Evolution*, 62: 777–92. 22138161 10.1016/j.ympev.2011.11.019 PMC3258026

[R6] Cornelissen M. et al. (2017) ‘From Clinical Sample to Complete Genome: Comparing Methods for the Extraction of HIV-1 RNA for High-Throughput Deep Sequencing’, *Virus**Research*, 239: 10–6. 10.1016/j.virusres.2016.08.004 27497916

[R7] Davanos N. et al. (2015) ‘HIV-1 Subtype Characteristics of Infected Persons Living in Southwestern Greece’, *HIV/AIDS* (*Auckland, N.Z.*), 7: 277–83. 26715861 10.2147/HIV.S90755 PMC4686321

[R8] Felsenstein J. (1981) ‘Evolutionary Trees From DNA Sequences: A Maximum Likelihood Approach’, *Journal of Molecular Evolution*, 17: 368–76. 7288891 10.1007/BF01734359

[R9] —— (2004) *Inferring Phylogenies*. Sunderland, MA: Sinauer.

[R10] Firth C. et al. (2010) ‘Using Time-Structured Data to Estimate Evolutionary Rates of Double-Stranded DNA Viruses’, *Molecular Biology and Evolution*, 27: 2038–51. 20363828 10.1093/molbev/msq088 PMC3107591

[R11] Gall A. et al. (2012) ‘Universal Amplification, Next-Generation Sequencing, and Assembly of HIV-1 Genomes’, *Journal of Clinical Microbiology*, 50: 3838–44. 22993180 10.1128/JCM.01516-12 PMC3502977

[R12] Gao F. et al. (1996) ‘The Heterosexual Human Immunodeficiency Virus Type 1 Epidemic in Thailand Is Caused by an Intersubtype (A/E) Recombinant of African Origin’, *Journal of Virology*, 70: 7013–29. 8794346 10.1128/jvi.70.10.7013-7029.1996 PMC190752

[R13] Gilbert M. T. et al. (2007) ‘The Emergence of HIV/AIDS in the Americas and Beyond’, *Proceedings of the National Academy of Sciences of the United States of America*, 104: 18566–70. 17978186 10.1073/pnas.0705329104 PMC2141817

[R14] Group, Insight Start Study et al. (2015) ‘Initiation of Antiretroviral Therapy in Early Asymptomatic HIV Infection’, *New England Journal of Medicine*, 373: 795–807. 26192873 10.1056/NEJMoa1506816 PMC4569751

[R15] Gryseels S. et al. (2020) ‘A Near Full-Length HIV-1 Genome From 1966 Recovered From Formalin-Fixed Paraffin-Embedded Tissue’, *Proceedings of the National Academy of Sciences of the United States of America*, 117: 12222–9. 32430331 10.1073/pnas.1913682117 PMC7275743

[R16] Hemelaar J. et al. (2011) ‘Global Trends in Molecular Epidemiology of HIV-1 during 2000–2007’, *AIDS*, 25: 679–89. 21297424 10.1097/QAD.0b013e328342ff93 PMC3755761

[R17] —— (2019) ‘Global and Regional Molecular Epidemiology of HIV-1, 1990–2015: A Systematic Review, Global Survey, and Trend Analysis’, *The**Lancet**Infectious Diseases*, 19: 143–55. 10.1016/S1473-3099(18)30647-9 30509777

[R18] International HIV Controllers Study . (2010) ‘The Major Genetic Determinants of HIV-1 Control Affect HLA Class I Peptide Presentation’, *Science*, 330: 1551–7. 21051598 10.1126/science.1195271 PMC3235490

[R19] Ishikawa S. A. et al. (2019) ‘A Fast Likelihood Method to Reconstruct and Visualize Ancestral Scenarios’, *Molecular Biology and Evolution*, 36: 2069–85. 31127303 10.1093/molbev/msz131 PMC6735705

[R20] Kozlov A. M., Aberer A. J., and Stamatakis A. (2015) ‘ExaML Version 3: A Tool for Phylogenomic Analyses on Supercomputers’, *Bioinformatics*, 31: 2577–9. 25819675 10.1093/bioinformatics/btv184 PMC4514929

[R21] Langmead B., and Salzberg S. L. (2012) ‘Fast Gapped-read Alignment with Bowtie 2’, *Nature**Methods*, 9: 357–9. 10.1038/nmeth.1923 PMC3322381 22388286

[R22] Letunic I., and Bork P. (2019) ‘Interactive Tree Of Life (iTOL) v4: Recent Updates and New Developments’, *Nucleic Acids**Research*, 47: W256–59. 10.1093/nar/gkz239 PMC6602468 30931475

[R23] Li H. et al. (2009) ‘The Sequence Alignment/Map Format and SAMtools’, *Bioinformatics*, 25: 2078–9. 19505943 10.1093/bioinformatics/btp352 PMC2723002

[R24] Liitsola K. et al. (1998) ‘HIV-1 Genetic Subtype A/B Recombinant Strain Causing an Explosive Epidemic in Injecting Drug Users in Kaliningrad’, *AIDS*, 12: 1907–19. 9792392 10.1097/00002030-199814000-00023

[R25] Melo F. L., Jamal L. F., and Zanotto P. M. (2012) ‘Characterization of Primary Isolates of HIV Type 1 CRF28_BF, CRF29_BF, and Unique BF Recombinants Circulating in Sao Paulo, Brazil’, *AIDS**Research and Human**Retroviruses*, 28: 1082–8. 10.1089/aid.2011.0123 PMC3423645 22176121

[R0025a] Molecular Biology and Evolution, Volume 36, Issue 9, September 2019, Pages 2069–2085.

[R26] Murphy E. et al. (1993) ‘Diversity of V3 Region Sequences of Human Immunodeficiency Viruses Type 1 From the Central African Republic’, *AIDS**Research and Human**Retroviruses*, 9: 997–1006. 10.1089/aid.1993.9.997 8280481

[R27] Neogi U. et al. (2012) ‘Molecular Epidemiology of HIV-1 Subtypes in India: Origin and Evolutionary History of the Predominant Subtype C’, *PLoS One*, 7: e39819. 10.1371/journal.pone.0039819 PMC3387228 22768132

[R28] —— (2017) ‘Recent Increased Identification and Transmission of HIV-1 Unique Recombinant Forms in Sweden’, *Scientific Reports*, 7: 6371. 10.1038/s41598-017-06860-2 PMC5527090 28744024

[R29] Ning Z., Cox A. J., and Mullikin J. C. (2001) ‘SSAHA: A Fast Search Method for Large DNA Databases’, *Genome**Research*, 11: 1725–9. 10.1101/gr.194201 PMC311141 11591649

[R30] Pineda-Pena A. C. et al. (2013) ‘Automated Subtyping of HIV-1 Genetic Sequences for Clinical and Surveillance Purposes: Performance Evaluation of the New REGA Version 3 and Seven Other Tools’, *Infection, Genetics and Evolution*, 19: 337–48. 10.1016/j.meegid.2013.04.032 23660484

[R31] Ratmann O. et al. (2017) ‘HIV-1 Full-Genome Phylogenetics of Generalized Epidemics in Sub-Saharan Africa: Impact of Missing Nucleotide Characters in Next-Generation Sequences’, *AIDS**Research and Human**Retroviruses*, 33: 1083–98. 10.1089/aid.2017.0061 PMC5597042 28540766

[R32] To T. H. et al. (2016) ‘Fast Dating Using Least-Squares Criteria and Algorithms’, *Systematic Biology*, 65: 82–97. 26424727 10.1093/sysbio/syv068 PMC4678253

[R33] Volz E. M., Koelle K., and Bedford T. (2013) ‘Viral Phylodynamics’, *PLoS**Computational Biology*, 9: e1002947. 10.1371/journal.pcbi.1002947 PMC3605911 23555203

[R34] Worobey M. et al. (2016) ‘1970s and “Patient 0” HIV-1 Genomes Illuminate Early HIV/AIDS History in North America’, *Nature*, 539: 98–101. 27783600 10.1038/nature19827 PMC5257289

