# Supplementary material for: Phylogenetic analysis of HIV-1 shows frequent cross-country transmission and local population expansions
Source: Virus Evol. 2021 Jun 9;7(2):veab055. doi: 10.1093/ve/veab055 (PMC8438898; doi:10.1093/ve/veab055)
Supplement: veab055_Supp [file veab055_supp.zip › Clean_supplementary_VEVOLU-2020-147_R4.docx]

**Supplementary tables and figures**

**s**

**Supplementary Table 1**: Primer sets used for reverse-transcription and PCR amplification of amplicon A and amplicon B.

| **Primer set** | **Sequence 5’-3’** | **HXB2 position (nt)** | **Product size** |
| --- | --- | --- | --- |
| Amplicon A forward | GGG AAG TGA YAT AGC WGG AAC | 1485-1505 | 3574 |
| Amplicon A reverse | CTG CCA TCT GTT TTC CAT ART C | 5058-5035 |  |
| Amplicon B forward | CCT ATG GCA GGA AGA AGC G | 5967-5987 | 3551 |
| Amplicon B reverse | CTT WTA TGC AGC WTC TGA GGG | 9517-9495 |  |

**Supplementary Table 2:** Distribution of subtypes per country.

| **Country** | **A** | **AB** | **CRF01_AE** | **B** | **BC** | **BF** | **C** | **Other** |
| --- | --- | --- | --- | --- | --- | --- | --- | --- |
| Argentina (n = 151) |  |  |  | 46% | 3% | 48% | 1% | 2% |
| Australia (n = 83) |  |  | 6% | 89% | 1% |  | 4% |  |
| Austria (n = 5) |  | 20% |  | 80% |  |  |  |  |
| Belgium(n = 88) | 1% | 1% | 2% | 72% |  | 3% | 7% | 14% |
| Brazil (n = 401) |  | 0% |  | 78% | 4% | 8% | 3% | 5% |
| Chile (n = 61) |  |  |  | 80% |  | 20% |  |  |
| Czech Republic (n=11) | 18% |  |  | 82% |  |  |  |  |
| Denmark (n = 29) |  |  | 10% | 76% |  |  | 3% | 10% |
| Estonia (n = 6) |  | 17% |  | 17% |  |  | 17% | 50% |
| Finland (n = 18) |  |  |  | 83% | 11% |  |  | 6% |
| France (n = 78) |  | 4% | 1% | 72% |  | 1% | 3% | 19% |
| Germany (n= 249) | 2% | 3% | 2% | 87% | 1% | 1% | 0% | 4% |
| Greece (n = 68) | 26% | 19% | 4% | 29% |  | 1% | 3% | 16% |
| India (n = 46) |  |  |  |  | 15% |  | 85% |  |
| Ireland (n = 4) |  |  |  | 75% |  |  | 25% |  |
| Israel (n = 14) | 14% |  |  | 71% | 7% |  | 7% |  |
| Italy (n = 27) |  | 7% |  | 74% |  | 11% | 4% | 4% |
| Luxembourg (n = 5) |  |  |  | 60% |  |  | 40% |  |
| Malaysia (n = 14) |  | 14% | 14% | 43% |  |  | 7% | 21% |
| Mali (n = 23) |  | 17% |  | 13% |  |  |  | 70% |
| Mexico (n = 38) |  |  |  | 100% |  |  |  |  |
| Morocco (n = 29) |  | 7% |  | 66% | 7% |  |  | 21% |
| Nigeria (n = 32) |  | 6% |  | 0% |  |  |  | 94% |
| Norway (n = 15) | 13% |  |  | 60% | 7% |  |  | 20% |
| Peru (n = 140) |  |  |  | 88% | 1% | 11% |  | 1% |
| Poland (n = 42) | 12% | 7% |  | 81% |  |  |  |  |
| Portugal (n = 55) | 7% | 5% |  | 56% |  | 5% | 5% | 20% |
| South Africa (n = 236) | 0% | 1% |  | 5% | 35% |  | 53% | 6% |
| Spain (n = 190) |  | 3% | 1% | 83% | 1% | 3% | 2% | 7% |
| Sweden (n = 2) |  |  | 50% |  |  | 50% |  |  |
| Switzerland (n = 26) | 4% |  | 4% | 81% |  | 4% |  | 8% |
| Thailand (n = 170) |  | 10% | 70% | 10% | 1% |  |  | 9% |
| Uganda (n = 231) | 27% | 14% |  | 4% | 0% |  | 2% | 53% |
| United Kingdom (n = 261) | 1% | 2% | 1% | 74% | 5% | 2% | 6% | 9% |
| United States (n = 349) |  | 1% |  | 97% | 0% | 0% | 1% | 1% |

**Supplementary Table 3**: Country distribution of LANL filtered web alignment samples

| **Country** | **CRF01_AE** | **A** | **B** | **C** | **D** | **F** | **G** |
| --- | --- | --- | --- | --- | --- | --- | --- |
| Afghanistan (n = 1) | 100% |  |  |  |  |  |  |
| Angola (n = 3) |  |  |  |  |  | 100% |  |
| Argentina (n = 30) |  |  | 93% | 3% |  | 3% |  |
| Australia (n = 19) |  | 5% | 95% |  |  |  |  |
| Belarus (n = 1) |  | 100% |  |  |  |  |  |
| Belgium (n = 2) |  |  |  |  |  | 50% | 50% |
| Bolivia (n = 1) |  |  | 100% |  |  |  |  |
| Botswana (n = 52) |  |  |  | 100% |  |  |  |
| Brazil (n = 230) |  |  | 84% | 7% | 1% | 7% |  |
| Cameroon (n = 32) | 3% | 13% |  |  | 13% | 28% | 44% |
| Canada (n = 8) |  |  | 100% |  |  |  |  |
| Central_African_Republic (n = 3) | 100% |  |  |  |  |  |  |
| Chad (n = 2) |  |  |  |  | 100% |  |  |
| China (n = 185) | 66% |  | 27% | 5% |  |  | 2% |
| Colombia (n = 4) |  |  | 100% |  |  |  |  |
| Congo_-_Kinshasa (n = 23) |  | 48% |  |  | 35% |  | 17% |
| Cuba (n = 9) |  |  | 67% |  |  |  | 33% |
| Cyprus (n = 90) |  | 21% | 67% | 10% | 1% | 1% |  |
| Denmark (n = 18) |  |  | 94% | 6% |  |  |  |
| Djibouti (n = 1) |  |  |  | 100% |  |  |  |
| Dominican_Republic (n = 4) |  |  | 100% |  |  |  |  |
| Ecuador (n = 2) |  |  | 100% |  |  |  |  |
| Ethiopia (n = 24) |  |  |  | 100% |  |  |  |
| Finland (n = 1) |  |  |  |  |  | 100% |  |
| France (n = 10) |  |  | 80% |  |  | 20% |  |
| Gabon (n = 1) |  |  | 100% |  |  |  |  |
| Georgia (n = 5) |  | 20% | 60% | 20% |  |  |  |
| Germany (n = 40) |  |  | 95% | 3% |  | 3% |  |
| Ghana (n = 1) |  |  |  |  |  |  | 100% |
| Guinea-Bissau (n = 2) |  |  | 50% |  |  |  | 50% |
| Haiti (n = 6) |  |  | 100% |  |  |  |  |
| Hong_Kong_SAR_China (n = 3) | 33% |  | 67% |  |  |  |  |
| India (n = 51) |  | 10% | 2% | 88% |  |  |  |
| Iran (n = 1) | 100% |  |  |  |  |  |  |
| Israel (n = 5) |  |  |  | 100% |  |  |  |
| Italy (n = 2) |  | 50% | 50% |  |  |  |  |
| Jamaica (n = 1) |  |  | 100% |  |  |  |  |
| Japan (n = 36) | 14% |  | 86% |  |  |  |  |
| Kenya (n = 74) |  | 85% |  | 7% | 4% |  | 4% |
| Malawi (n = 25) |  |  |  | 100% |  |  |  |
| Myanmar_(Burma) (n = 4) | 50% |  | 25% | 25% |  |  |  |
| Nepal (n = 8) |  |  |  | 100% |  |  |  |
| Netherlands (n = 3) |  |  | 100% |  |  |  |  |
| Nigeria (n = 30) |  |  |  | 3% |  |  | 93% |
| Pakistan (n = 16 |  | 94% |  | 6% |  |  |  |
| Paraguay (n = 3) |  |  | 100% |  |  |  |  |
| Peru (n = 15) |  |  | 100% |  |  |  |  |
| Philippines (n = 17) | 76% |  | 24% |  |  |  |  |
| Poland (n = 1) |  |  | 100% |  |  |  |  |
| Portugal (n = 4) |  |  |  |  |  |  | 100% |
| Romania (n = 2) |  |  |  |  |  | 100% |  |
| Russia (n = 27) |  | 70% | 26% |  |  |  | 4% |
| Rwanda (n = 9) |  | 100% |  |  |  |  |  |
| Senegal (n = 6) |  | 50% |  | 33% | 17% |  |  |
| Somalia (n = 1) |  |  |  | 100% |  |  |  |
| South_Africa (n = 354) |  | 1% | 3% | 95% | 1% |  |  |
| South_Korea (n = 28) |  |  | 100% |  |  |  |  |
| Spain (n = 76) |  | 4% | 74% | 5% |  | 8% | 9% |
| Sweden (n = 88) | 7% | 11% | 26% | 53% | 1% |  | 1% |
| Switzerland (n = 15) |  | 7% | 93% |  |  |  |  |
| Taiwan (n = 1) |  |  | 100% |  |  |  |  |
| Tanzania (83 |  | 29% |  | 69% | 2% |  |  |
| Thailand (n = 140) | 82% |  | 18% |  |  |  |  |
| Trinidad_&_Tobago (n = 4) |  |  | 100% |  |  |  |  |
| Uganda (n = 54) |  | 33% |  |  | 67% |  |  |
| Ukraine (n =17) |  | 82% | 18% |  | 0% |  |  |
| United_Kingdom (n = 113) | 4% | 4% | 58% | 26% | 4% | 1% | 3% |
| United_States (n = 447) | 1% |  | 98% | 1% |  |  |  |
| Uruguay (n = 5) |  |  | 80% | 20% |  |  |  |
| Vietnam (n = 11) | 100% |  |  |  |  |  |  |
| Yemen (n = 5) |  |  | 40% | 20% | 40% |  |  |
| Zambia (n = 29) |  |  |  | 100% |  |  |  |
| NA (n = 8) |  |  | 63% | 13% |  | 13% | 13% |

**Supplementary Figure 1**: Histogram of the number of samples per for START (n=2,501) and LANL Filtered Web Alignment (n=2,632), respectively.

**Supplementary Figure 2:** Monophyletic clade for subtype B extracted from maximum likelihood phylogenetic tree of all samples (Figure 1). The outer color-strip indicate if the samples are from either START (white) or LANL Filtered Web Alignment (red) dataset.

**Supplementary Figure 3:** Monophyletic clade for subtype C extracted from maximum likelihood phylogenetic tree of all samples (Figure 1). The outer color-strip indicate if the samples are from either START (white) or LANL Filtered Web Alignment (red) dataset.

**Supplementary Figure 4:** Monophyletic clade for subtype A extracted from maximum likelihood phylogenetic tree of all samples (Figure 1). The outer color-strip indicate if the samples are from either START (white) or LANL Filtered Web Alignment (red) dataset.

**Supplementary Figure 5:** Monophyletic clade for subtype CRF01_AE extracted from maximum likelihood phylogenetic tree of all samples (Figure 1). The outer color-strip indicate if the samples are from either START (white) or LANL Filtered Web Alignment (red) dataset.

**Supplementary Figure 6:** Ancestral state reconstruction of subtype B samples with country state randomly shuffled.

**Supplementary Figure 7:** Ancestral state reconstruction of subtype C samples with country state randomly shuffled.

**Supplementary Figure 8:** Ancestral state reconstruction of subtype A samples with country state randomly shuffled.

**Supplementary Figure 9:** Ancestral state reconstruction of subtype CRF01_AE samples with country state randomly shuffled.
