# Supplementary figures and images for: Phylogenetic analysis of HIV-1 shows frequent cross-country transmission and local population expansions
Source: Virus Evol. 2021 Jun 9;7(2):veab055. doi: 10.1093/ve/veab055 (PMC8438898; doi:10.1093/ve/veab055)

Temporal distribution samples: Number of samples per year

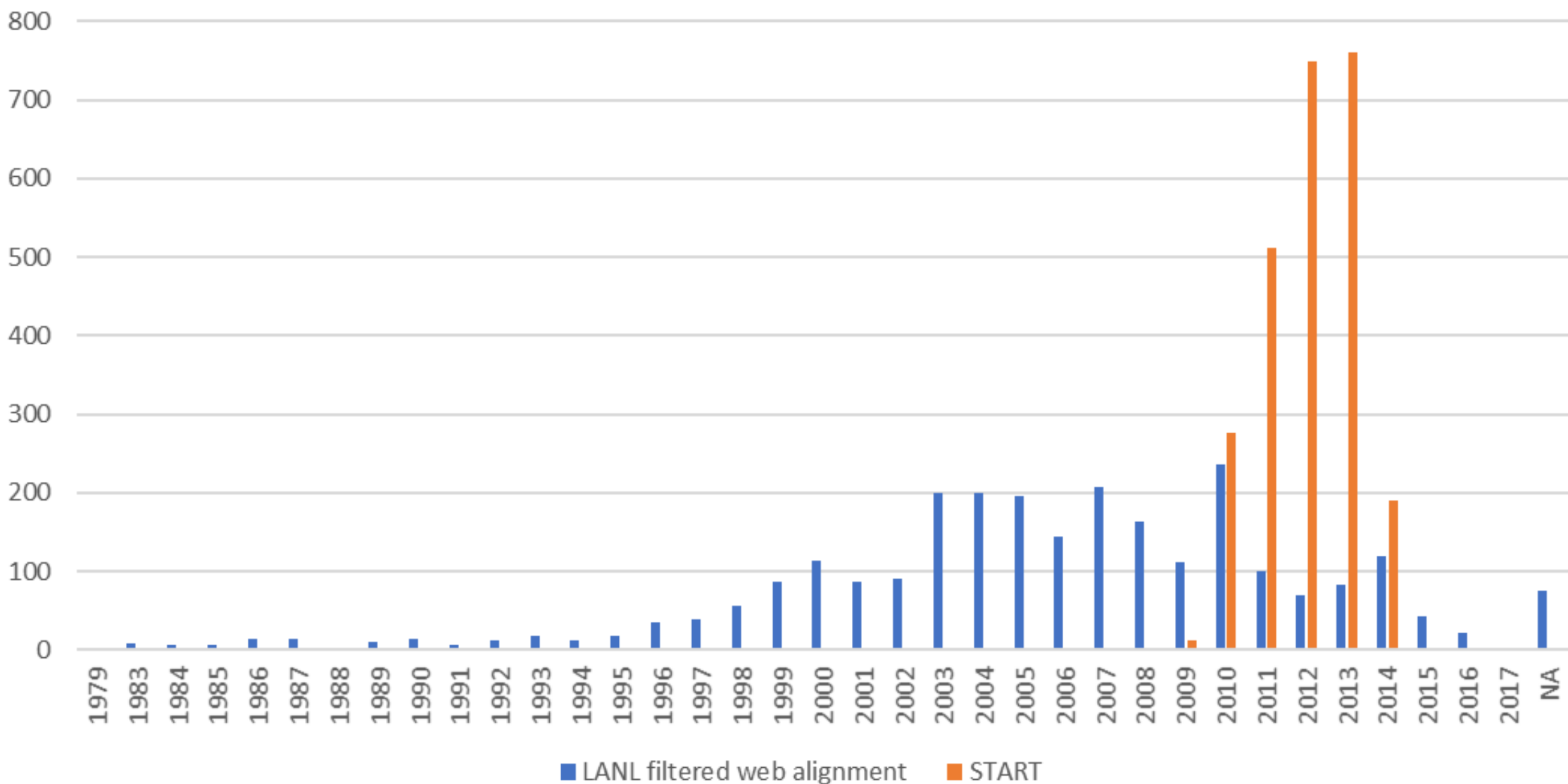

Supplement: veab055_Supp [file veab055_supp.zip › Supplementary_Figure_1_temporal_distribution.pdf]

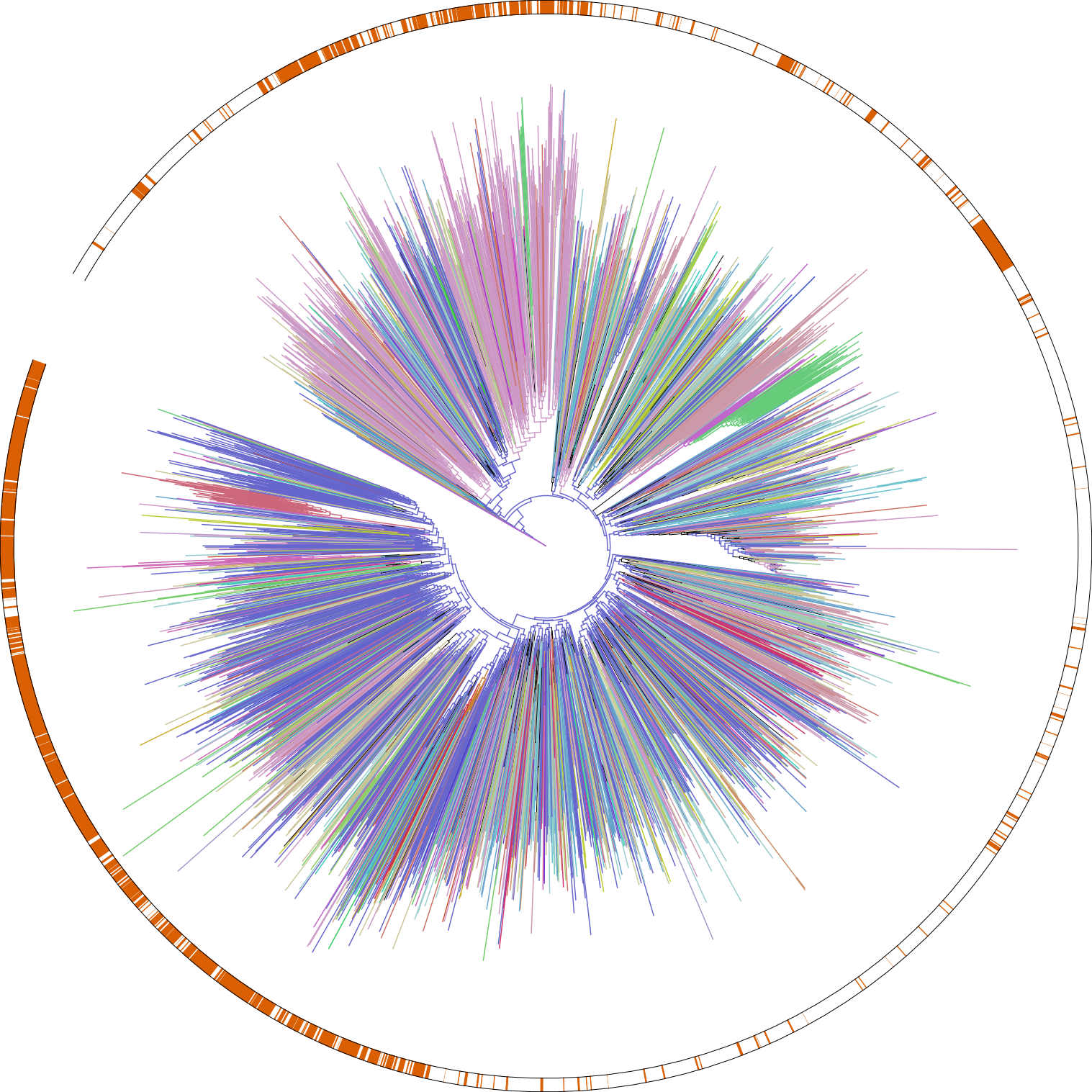

Supplement: veab055_Supp [file veab055_supp.zip › Supplementary_Figure_2_B_start_lanl_tree.pdf]

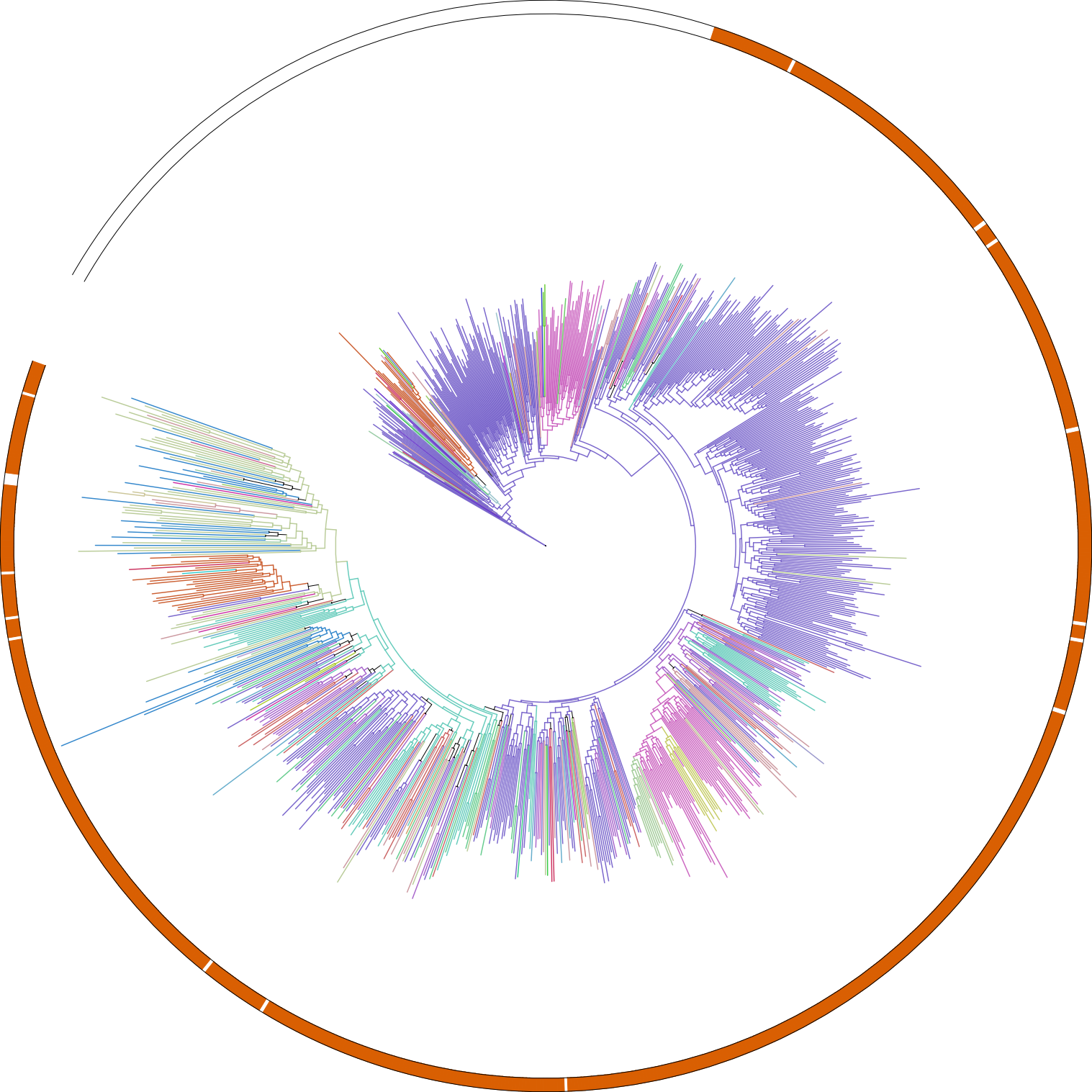

Supplement: veab055_Supp [file veab055_supp.zip › Supplementary_Figure_3_C_start_lanl_tree.pdf]

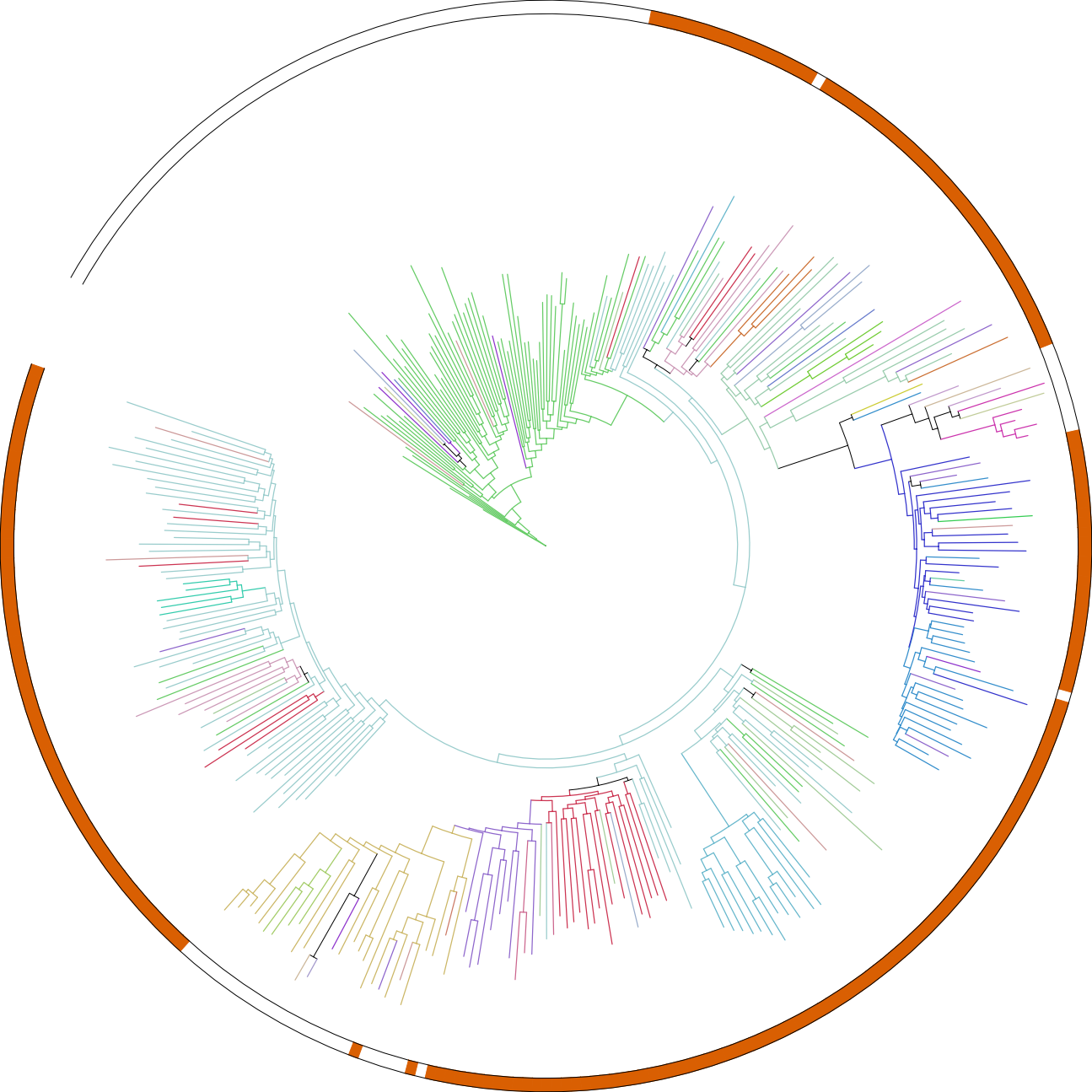

Supplement: veab055_Supp [file veab055_supp.zip › Supplementary_Figure_4_A_start_lanl_tree.pdf]

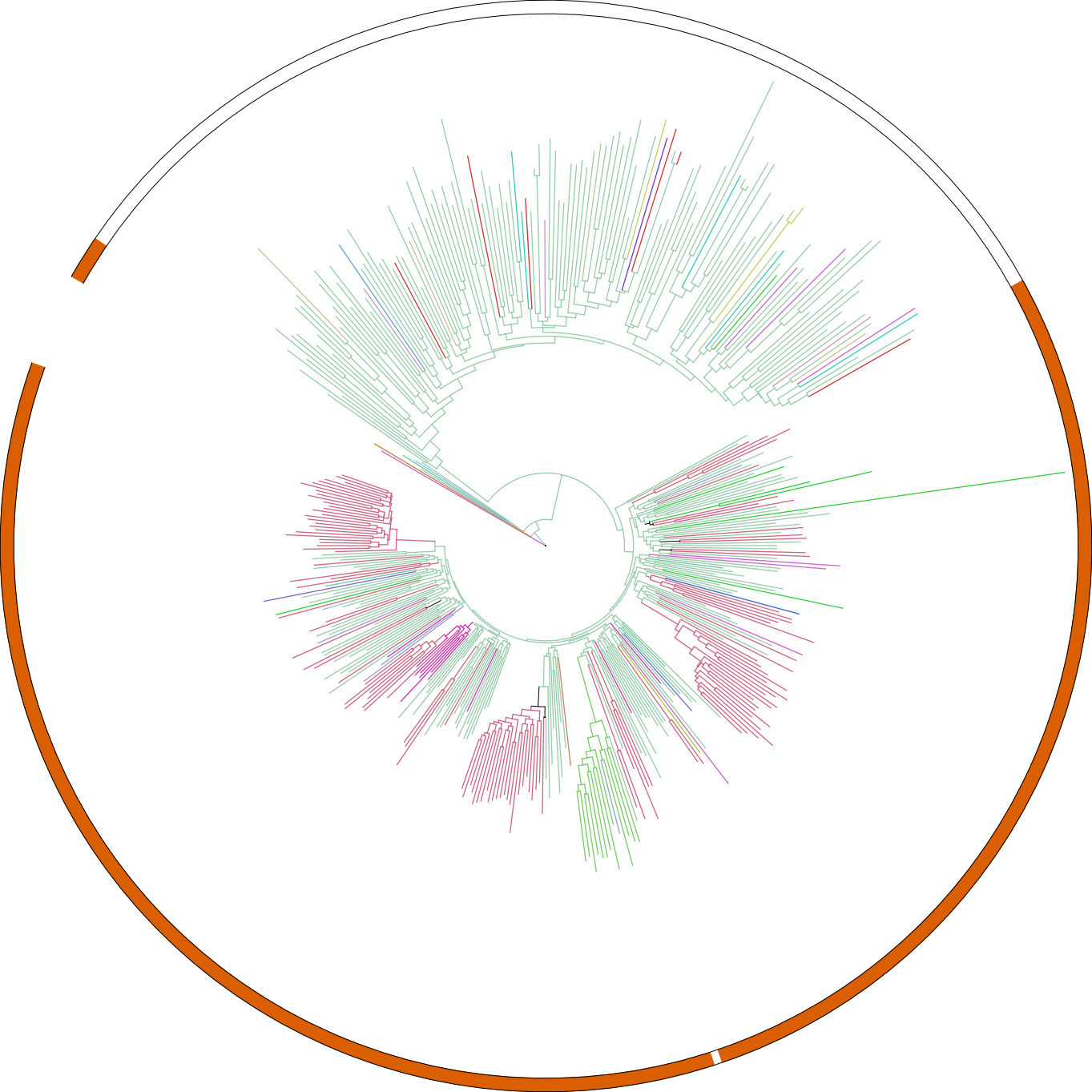

Supplement: veab055_Supp [file veab055_supp.zip › Supplementary_Figure_5_CRF01_AE_start_lanl_tree.pdf]
