# Supplementary material for: Phylogenetic analysis of HIV-1 shows frequent cross-country transmission and local population expansions
Source: Virus Evol. 2021 Jun 9;7(2):veab055. doi: 10.1093/ve/veab055 (PMC8438898; doi:10.1093/ve/veab055)
Supplement: veab055_Supp [file Supplementary_Figure_6_B_country_shuffled.pdf › Supplementary_Figure_6_B_country_shuffled.pdf]

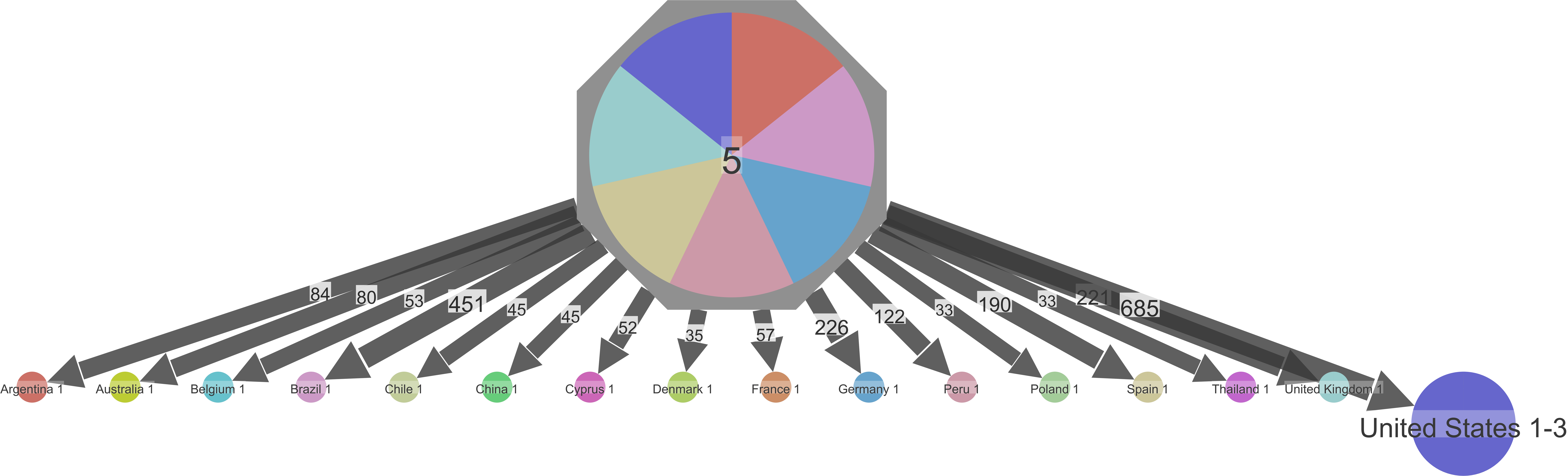

5
84
80
53
451
45
45
52
35
57
226
122
33
190
33
221
685
Argentina 1
Australia 1
Belgium 1
Brazil 1
Chile 1
China 1
Cyprus 1
Denmark 1
France 1
Germany 1
Peru 1
Poland 1
Spain 1
Thailand 1
United Kingdom 1
United States 1-3
