# Supplementary material for: Phylogenetic analysis of HIV-1 shows frequent cross-country transmission and local population expansions
Source: Virus Evol. 2021 Jun 9;7(2):veab055. doi: 10.1093/ve/veab055 (PMC8438898; doi:10.1093/ve/veab055)
Supplement: veab055_Supp [file Supplementary_Figure_7_C_country_shuffled.pdf › Supplementary_Figure_7_C_country_shuffled.pdf]

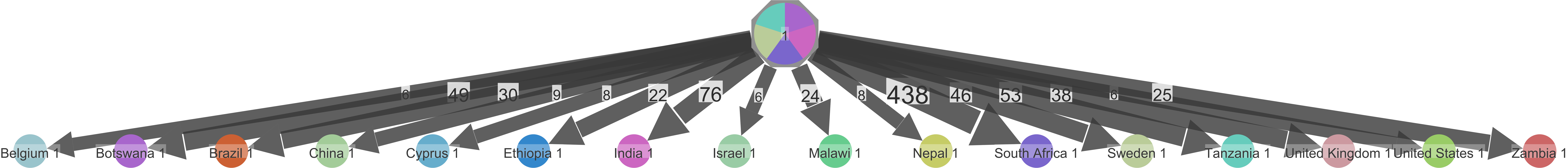
1
6
49
30
9
8
22
76
6
24
8
438
46
53
38
6
25
Belgium 1
Botswana 1
Brazil 1
China 1
Cyprus 1
Ethiopia 1
India 1
Israel 1
Malawi 1
Nepal 1
South Africa 1
Sweden 1
Tanzania 1
United Kingdom 1
United States 1
Zambia 1
