# Supplementary material for: Phylogenetic analysis of HIV-1 shows frequent cross-country transmission and local population expansions
Source: Virus Evol. 2021 Jun 9;7(2):veab055. doi: 10.1093/ve/veab055 (PMC8438898; doi:10.1093/ve/veab055)
Supplement: veab055_Supp [file Supplementary_Figure_8_A_country_shuffled.pdf › Supplementary_Figure_8_A_country_shuffled.pdf]

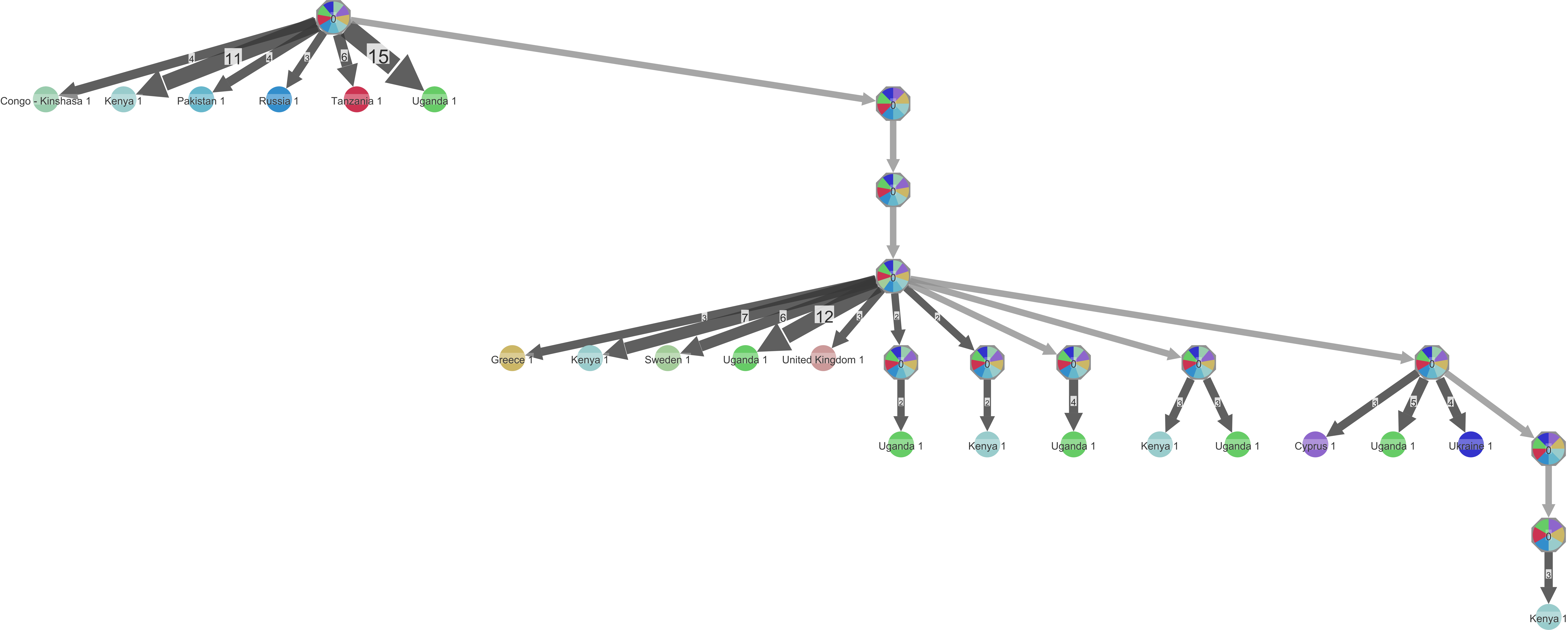

0
4
11
4
3
6
15
Congo - Kinshasa 1
Kenya 1
Pakistan 1
Russia 1
Tanzania 1
Uganda 1
0
0
0
3
7
6
12
3
2
2
Greece 1
Kenya 1
Sweden 1
Uganda 1
United Kingdom 1
0
0
0
0
0
2
2
4
3
3
3
5
4
Uganda 1
Kenya 1
Uganda 1
Kenya 1
Uganda 1
Cyprus 1
Uganda 1
Ukraine 1
0
0
3
Kenya 1
