# Supplementary material for: Phylogenetic analysis of HIV-1 shows frequent cross-country transmission and local population expansions
Source: Virus Evol. 2021 Jun 9;7(2):veab055. doi: 10.1093/ve/veab055 (PMC8438898; doi:10.1093/ve/veab055)
Supplement: veab055_Supp [file Supplementary_Figure_9_CRF01_AE_country_shuffled.pdf › Supplementary_Figure_9_CRF01_AE_country_shuffled.pdf]

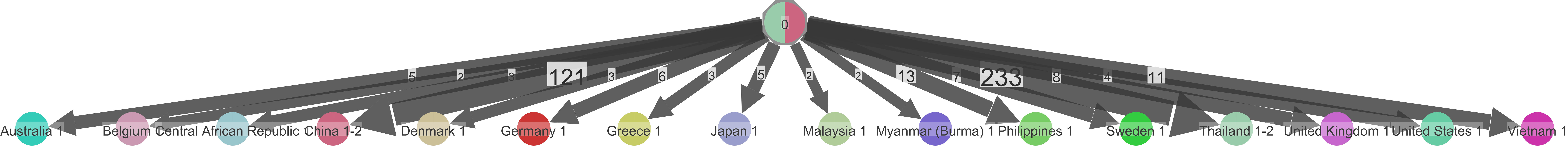

0
5
2
3
121
3
6
3
5
2
2
13
7
233
8
4
11
Australia 1
Belgium 1
Central African Republic 1
China 1-2
Denmark 1
Germany 1
Greece 1
Japan 1
Malaysia 1
Myanmar (Burma) 1
Philippines 1
Sweden 1
Thailand 1-2
United Kingdom 1
United States 1
Vietnam 1
